# Integrating Machine Learning for Predictive Maintenance on Resource-Constrained PLCs: A Feasibility Study

**DOI:** 10.3390/s25020537

**Published:** 2025-01-17

**Authors:** Riccardo Mennilli, Luigi Mazza, Andrea Mura

**Affiliations:** Department of Mechanical and Aerospace Engineering, Politecnico di Torino, 10129 Turin, Italy; riccardo.mennilli@studenti.polito.it (R.M.); andrea.mura@polito.it (A.M.)

**Keywords:** PLC, Arduino board, industrial automation, edge computing, machine learning, predictive maintenance, structural health monitoring

## Abstract

This study investigates the potential of deploying a neural network model on an advanced programmable logic controller (PLC), specifically the Finder Opta™, for real-time inference within the predictive maintenance framework. In the context of Industry 4.0, edge computing aims to process data directly on local devices rather than relying on a cloud infrastructure. This approach minimizes latency, enhances data security, and reduces the bandwidth required for data transmission, making it ideal for industrial applications that demand immediate response times. Despite the limited memory and processing power inherent to many edge devices, this proof-of-concept demonstrates the suitability of the Finder Opta™ for such applications. Using acoustic data, a convolutional neural network (CNN) is deployed to infer the rotational speed of a mechanical test bench. The findings underscore the potential of the Finder Opta™ to support scalable and efficient predictive maintenance solutions, laying the groundwork for future research in real-time anomaly detection. By enabling machine learning capabilities on compact, resource-constrained hardware, this approach promises a cost-effective, adaptable solution for diverse industrial environments.

## 1. Introduction

Industry 4.0 refers to the integration of traditional industrial processes with smart digital environments, aiming to collect vast amounts of data from every stage of the manufacturing cycle. This digital revolution enhances the speed of information exchange and enables data-driven advantages such as cost reduction, lower downtime, and improved operator safety [1,2,3]. One of the most promising technologies in this context is machine learning (ML), especially as it applies to predictive maintenance, where ML algorithms are used to predict failures and optimize machinery efficiency [4,5,6].

Machine learning, particularly artificial neural networks (ANNs), can handle complex, high-dimensional data, extracting hidden relationships from various sources to become a valuable predictive tool in manufacturing [7]. However, deploying ML models in traditional production environments is challenging due to high computational costs and the need for centralized infrastructure, which demands significant investments [8]. A key strategy to address these challenges is edge computing, which involves deploying ML models directly on terminal devices like programmable logic controllers (PLCs), eliminating the need for large cloud infrastructures. This approach enhances processing speed, privacy, and cost-efficiency [9].

By processing data directly on devices like PLCs, edge computing allows for real-time applications such as rapid fault detection, which would otherwise be impossible due to the latency inherent in cloud-based architectures [10,11]. However, limitations in memory and power still represent a major challenge for the development of edge computing, especially when machine learning is involved. Many hybrid architectures are being investigated to circumvent the issue, such as training the model on the cloud before deploying it to the edge device, or “Federated Learning”, proposed by Google in 2016 [12], where deep learning models are trained at the edge, with the cloud serving as a model aggregator. The former approach is the most common and straightforward and will be employed in this study, though it does present some drawbacks, especially when the model needs updating since all of the data need to be transferred to and from the cloud.

Predictive maintenance is one of the fields where machine learning can be most impactful [13,14,15]. It represents a middle ground between “Run-to-Failure” strategies, where maintenance is performed only after machinery fails, and “Preventive Maintenance”, scheduled at regular time intervals regardless of the actual condition of the equipment [16,17]. Instead, by continuously monitoring a machine’s condition, predictive maintenance enables companies to anticipate breakdowns and perform maintenance only when needed, accurately estimating the Remaining Useful Life (RUL) and minimizing unexpected downtimes [18]. This approach has been shown to improve overall equipment effectiveness by over 90% [19].

Among the various ML methods, ANNs stand out due to their ability to operate in real-time and their robustness against inconsistent data and outliers. ANNs do not require domain-specific knowledge, making them applicable across various industries [20]. However, one major disadvantage is the significant cost involved in training and executing these models, as they require large amounts of data and computing resources [21]. Research in this area focuses on reducing the computational footprint of these models to make them suitable for deployment in resource-constrained environments like edge devices.

An important aspect of predictive maintenance discussed in this paper is the use of acoustic data for anomaly detection. Anomalous sound detection is an emerging field that involves identifying abnormal sounds emitted by machinery to detect early signs of failure [22]. Traditionally, skilled technicians could identify such anomalies by ear, but this approach lacks scalability [23]. The use of AI for sound-based anomaly detection is a promising alternative, allowing for the automation of this process [24]. For example, AI-based sound detection has been successfully applied to fault detection in vertical drilling machines [25], with current research also focusing on unsupervised models to recognize abnormal situations without any a priori knowledge [26,27].

The case study presented in the paper explores the deployment of an ANN model to infer the rotational speed of ball bearings using acoustic data recorded by an affordable electret microphone. Although the case study primarily focuses on speed measurement, the broader goal is predictive maintenance within Industry 4.0, where acoustic data are used to detect equipment malfunction. The rotational speed measurement was selected as an initial test case due to its simplicity in acquiring measurable signals from basic sensors, thereby enabling efficient model training and facilitating a clear comparison between predicted and actual values. However, it does not represent the ultimate objective, as future iterations of this work aim to contribute to predictive maintenance strategies within the framework of Industry 4.0, leveraging acoustic data to detect potential equipment malfunctions.

The adaptability of this system for retrofitting existing processes with minimal cost could foster adoption rates even in smaller plants or where it would not make financial sense to employ high-end solutions because of the type of technology (i.e., pneumatic systems that are known for their reliability, making fault detection critical, but low overall costs).

This work serves as a preliminary proof of concept aimed at evaluating whether an innovative PLC device with Arduino integration, such as the Finder Opta™, can support real-time inference with an artificial neural network. Nowadays, several solutions to implement machine learning algorithms and AI are available on the PLC market, with manufacturers providing dedicated modules, programmable, for example, in C++ or MicroPython. These, however, are often quite costly and do not offer the flexibility and ease of use of the Arduino platform, which also has the advantage of being open source. The Finder Opta™ was, therefore, deemed an appropriate choice for this work in order to build an affordable and easy-to-retrofit PLC system with a low entry barrier. A more exhaustive comparative analysis against alternatives on the market is out of the scope of this feasibility study and will be carried out in future work.

By demonstrating initial feasibility in tasks such as rotational speed measurement from acoustic data, this study lays the groundwork for more advanced applications.

### Paper Structure

Section 2 focuses on the experimental setup and hardware configuration, with a description of the key features of the Finder Opta™ PLC that enable research of this kind. Section 3 goes into detail about the methodology and the case study, illustrating the main steps of data acquisition, data processing, model architecting, and model deployment. Section 4 presents the experimental results, along with their critical analysis, and Section 5 presents the conclusions, including future developments.

## 2. Experiment Setup

The experimental test bench is designed to evaluate the Finder Opta™ PLC’s ability to perform real-time speed inference through embedded neural networks using acoustic data.

**Figure 1:**
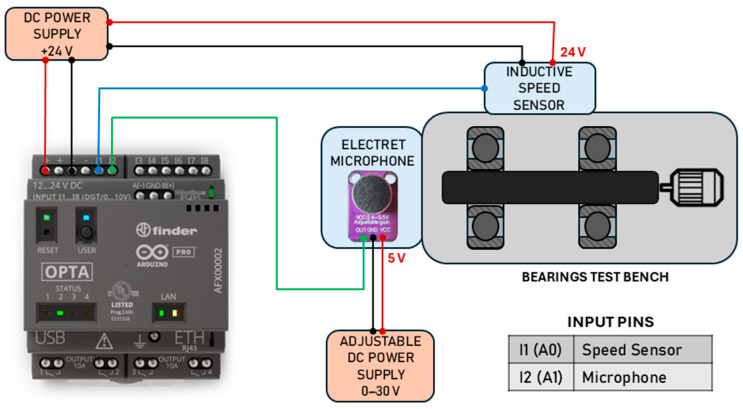
Experiment setup diagram.

**Figure 2:**
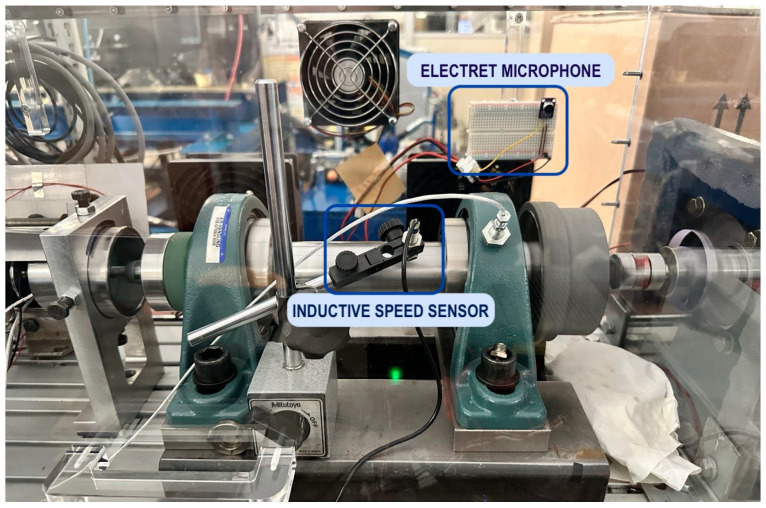
Experiment setup.

The setup (Figure 1 and Figure 2) consists of a rotating shaft driven by an asynchronous electric motor, supported at each end by radial ball bearings. Originally developed for the characterization of bearing lubrication, this test bench highlights the adaptability of the proposed approach for retrofitting into existing systems. The motor’s speed is managed through a closed-loop PID control system, which is tuned to minimize fluctuation and maintain stable rotations within a specified range (200–1500 rpm). The motor is powered by an inverter, which links to a Human–Machine Interface (HMI) for easy speed monitoring and adjustments.

The two primary sensors deployed in this experiment are an electret microphone, the ARCELI GY-MAX4466, and an inductive proximity sensor. The electret microphone captures the sound emitted by the rotating shaft and bearings. Acoustic monitoring was selected because sound analysis is highly effective in detecting mechanical anomalies, such as misalignments or bearing wear, which can manifest as deviations in sound patterns.

The microphone’s output is pre-amplified to enhance signal quality and ensure compatibility with the Finder Opta’s analog-to-digital converter (ADC), which accepts signals within a 0–10 V range. This configuration eliminates the need for additional custom amplification, streamlining integration while maintaining adequate sensitivity.

The inductive proximity sensor, powered at 24 V DC, is mounted near the rotating shaft and detects the passages of a small magnet attached to the shaft. This sensor provides precise speed measurements by counting the magnetic pulses over a defined interval, yielding accurate speed labels essential for supervised training and model validation. The resulting data pairs—acoustic signals and corresponding speed labels—form the neural network’s training dataset.

### The Finder Opta™ PLC

The core of this study is the Finder Opta™ PLC, a compact and versatile Programmable Logic Controller designed through a collaboration between Finder and Arduino. This device incorporates a dual-core ARM Cortex-M processor (ST STM32H747XI, STMicroelectronics), making it suitable for applications requiring both high performance and resource efficiency. The Finder Opta’s hardware architecture, coupled with its industrial-grade form factor, supports a wide range of connectivity options, including Wi-Fi, Bluetooth LE, RS-485, as well as Ethernet for robust communication capabilities in industrial environments focused on IoT applications.

A key advantage of the Finder Opta™ in this context is its compatibility with the Arduino ecosystem, allowing it to be programmed with both traditional PLC languages (IEC 61131-3) and the Arduino variant of C++. This dual compatibility significantly lowers the entry barrier for adopting state-of-the-art technologies, making advanced machine learning and IoT applications accessible even to technicians without extensive experience in the field. The Arduino programming environment provides access to countless official and third-party libraries, such as TensorFlow Lite for Arduino, which is the machine learning framework of choice employed in this work, enabling rapid development and easy integration of multiple technologies. Note, however, that at the time of writing, the official version of the library (2.4.0-ALPHA) only supports the Arduino Nano 33 BLE Sense board. Nevertheless, it is possible to eliminate the explicit references to the Nano board peripherals, as the framework code is compatible with most Arm Cortex M-based boards, including the Finder Opta™.

The official Arduino IDE (version 2.3) is used to develop the software to run on the Opta™, accessed via the serial port over USB.

## 3. Methods and Case Study

### 3.1. Data Collection and Preprocessing

**Figure 3:**
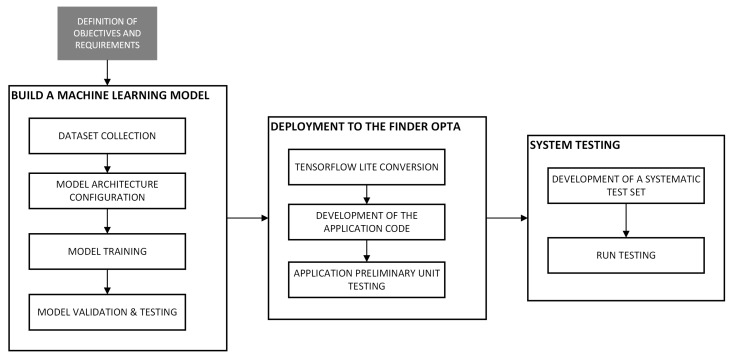
Machine learning workflow.

The workflow, depicted in Figure 3, starts with the data collection phase, which involves recording the sound emitted by the test bench in operation, exploiting the Opta’s analog inputs to sample the output voltage from the microphone. A sampling frequency of 5000 Hz allows capturing the relevant frequencies while addressing the PLC’s memory constraints, as it reduces the number of data points required to store a recording of significant duration.

The inductive proximity sensor provides an accurate rotational speed measurement, producing speed labels necessary for supervised learning. To build a comprehensive dataset, 1350 audio samples are recorded across the relevant range of speeds (200–1500 rpm, with 50-rpm increments) and under various operational conditions at different times of the day to account for different levels of foreign noise present on the laboratory floor.

The raw voltage signals undergo on-device preprocessing to extract higher-level features. While it would be conceivable to feed the model the raw voltage signal sampled by the PLC, extracting meaningful features would pose quite a big challenge for a small network. Instead, a common approach in audio recognition is providing a spectrogram as input, i.e., a 2D representation of the frequency spectrum of the signal over time, a higher-layer abstraction with the most useful information [28].

**Figure 4:**
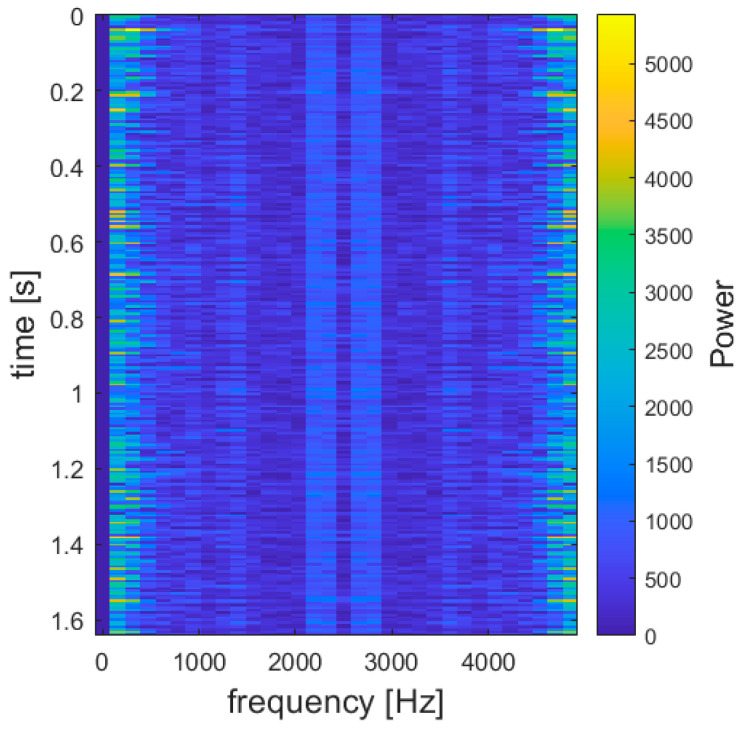
Spectrogram example—900 rpm.

The Fast Fourier Transform (FFT) algorithm is applied to the segments of the audio signal to build their spectrograms, thanks to the ArduinoFFT library (v 2.0.2). Spectrograms offer a 2D matrix format, with one axis representing time and the other frequency (Figure 4). This format allows the neural network to recognize both temporal and frequency-domain patterns, which are crucial for distinguishing between different rotational speeds. The spectrogram dimensions were set to 256 × 32, a trade-off between information density and their size. This results in a 1.64 s long audio acquisition process.

### 3.2. Model Architecture and Training

A regular Multilayer Perceptron model is not suitable for audio or image recognition because of its inability to work with multidimensional tensors and recognize the relationships between groups of adjacent pixels. Instead, *convolutional neural networks (CNNs)* were developed precisely for such purpose, as they can learn how simple features of a multidimensional tensor fit together into more complex structures, aiding in the interpretation of the frequency information contained in the spectrogram. A CNN is, therefore, the most natural choice here [28,29].

**Figure 5:**
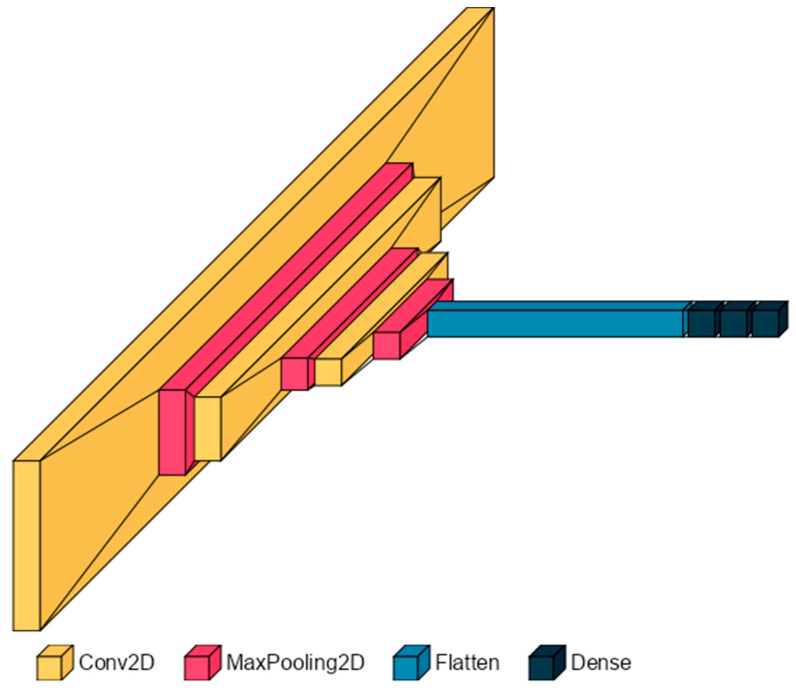
CNN Architecture.

**Figure 6:**
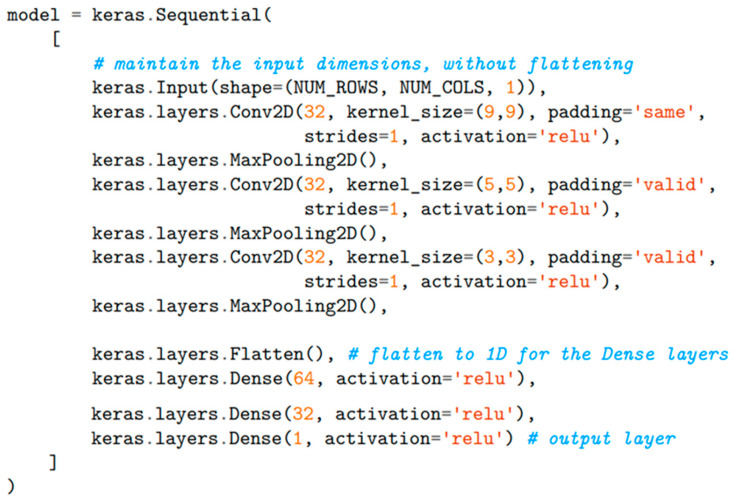
CNN Model Design in TensorFlow, using Python in Google Colab.

The CNN architecture (Figure 5) can be constructed with the Sequential Keras API (Figure 6) since only a single input and output tensor are required for each layer. The input to the model is a two-dimensional 32 × 256 tensor, while the output layer comprises a single neuron whose activation represents the speed prediction, as this is a regression problem.

One of the most common activation functions nowadays is the ReLU or rectified linear unit. This is especially true for CNNs, where their low computational complexity and cleanly defined gradients provide significant advantages in terms of training speed and accuracy.

The ReLU is, therefore, the choice for all layers of the network, including the output, where it is particularly appropriate since it leaves positive speeds unaltered (*y* = *x*, for *x* ≥ 0) and brings negative predictions, which are unphysical, to zero. Three 2D convolutional layers are employed to extract the high-level features from the spectrograms. Each layer defined with the *keras.layers.Conv2D()* class is composed of 32 filters for the convolution, striding one pixel at a time. The filter size is decreased deeper into the network for improved accuracy and to maintain compatible dimensions with the layers as they are down-sampled by three *MaxPooling2D()* operations that halve the size of the input. The structure is completed with two hidden fully connected layers of 64 and 32 nodes, respectively, that predict the speed on the basis of the features extracted by the convolutional layers.

**Figure 7:**
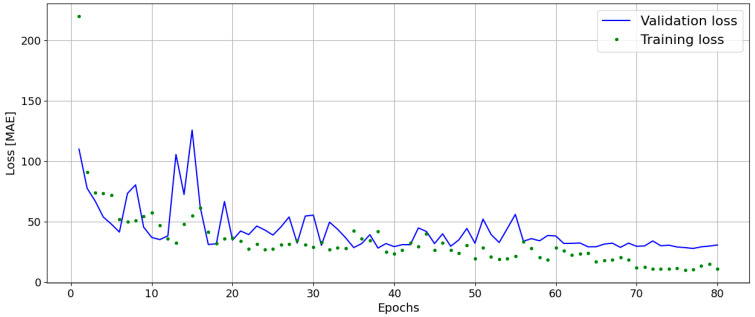
Training and validation loss.

Model training is performed in *Google Colab* using *TensorFlow (version 2.15.1)*, leveraging GPU acceleration to optimize learning speed. The collected dataset is randomized and split into training, validation, and test sets, with a 70%/15%/15% ratio, to evaluate and optimize model performance. This is carried out in *Google Colab* with the Python library *numpy.* For loss calculation, the Mean Absolute Error (MAE) metric (Equation (1), with n the number of predictions, with $y_{i}$ being the true outputs and ${\hat{y}}_{i}$ being the predicted ones) is chosen due to its robustness against outliers that may arise from extraneous noise in the acoustic data. Moreover, MAE provides a more balanced view, avoiding the disproportionate weighting of outliers that may arise from noise in acoustic data [30]. This choice aligns well with predictive maintenance objectives, where robustness to noise and accuracy are prioritized. An adaptive learning rate scheduler is implemented to adjust the learning rate dynamically [31], allowing the model to converge more efficiently on a (sub-)optimal solution (Figure 7).

The trained model is validated on the test set, achieving an average prediction error of 28.7 rpm. The final CNN configuration represents a balance between predictive accuracy and computational efficiency, making it suitable for real-time deployment on the Finder Opta™ with its memory constraints.

(1) $$\mathrm{MAE}\;(\mathrm{Mean}\;\mathrm{Absolute}\;\mathrm{Error})=\frac{1}{n}\sum_{i=1}^{n}\left|y_{i}-{\hat{y}}_{i}\right|,$$

### 3.3. Model Optimization and Deployment with TensorFlow Lite

Deploying the trained convolutional neural network to the Finder Opta™ PLC requires conversion to the TensorFlow Lite format for size reduction and optimization. Alongside this process, the embedded application to acquire the audio samples, compute their spectrograms, and run inference with the model needs to be designed.

TensorFlow is primarily thought for desktop and cloud environments and does not address properly the confinements of embedded applications of machine learning. For this reason, the *TensorFlow Lite* (TFLite) project was initiated in 2017, enabling much smaller binary sizes by eliminating certain features, such as the ability to train models directly on target devices or dropping support for more complex architectures [32]. This opened the door to experimentation with machine learning on small, mobile, memory-constrained devices, bringing advanced inference capabilities closer to the end application.

Thanks to the TensorFlow Lite Converter’s Python API, the model can be encoded as a *FlatBuffer* [33], applying post-training full-integer quantization to achieve a smaller footprint in terms of memory and resource utilization.


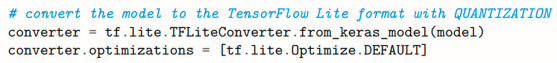


Full-integer quantization requires a representative dataset to estimate the typical range of variation of the variables involved. To this end, it is convenient to provide the converter with the test dataset, as it contains a sufficient number of examples to completely characterize the input.


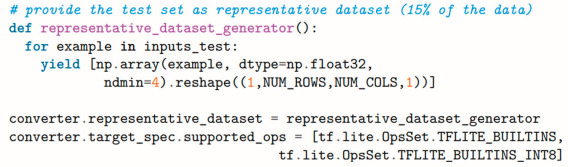


Lastly, int8 (fixed-point 8-bit integer) is specified as the target datatype for all internal quantities, while float32 is still used for the input and output tensors. This ensures better future compatibility with traditional applications, as float32 is the most common type for tensors, and the Finder Opta is not limited to integer-only operations.


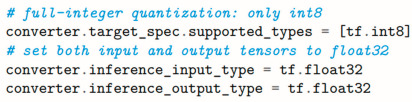


The model can be finally converted with the specified optimization and saved as a C byte array to be embedded in an Arduino header file. The resulting size is 1029 kB, which leaves just enough memory available for the rest of the code.


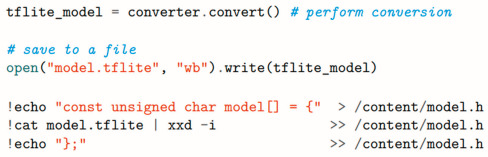


Once deployed, the Finder Opta™ captures the audio in real-time from the test bench via the electret microphone. The signal is processed on-device to produce spectrograms that match the input format used during model training. These are fed to the CNN model to conduct inference and obtain a speed prediction. Given the potential for foreign noise in industrial environments, the predicted speeds are averaged over a 10-s interval, mitigating the effects of temporary fluctuations and environmental noise-enhancing robustness.

## 4. Results and Discussion

The deployment of the convolutional neural network (CNN) model on the Finder Opta™ PLC for real-time rotational speed inference yielded a promising outcome, validating the feasibility of integrating machine learning applications within resource-constrained industrial devices. Table 1 and Table 2 report the experimental results.

**Figure 8:**
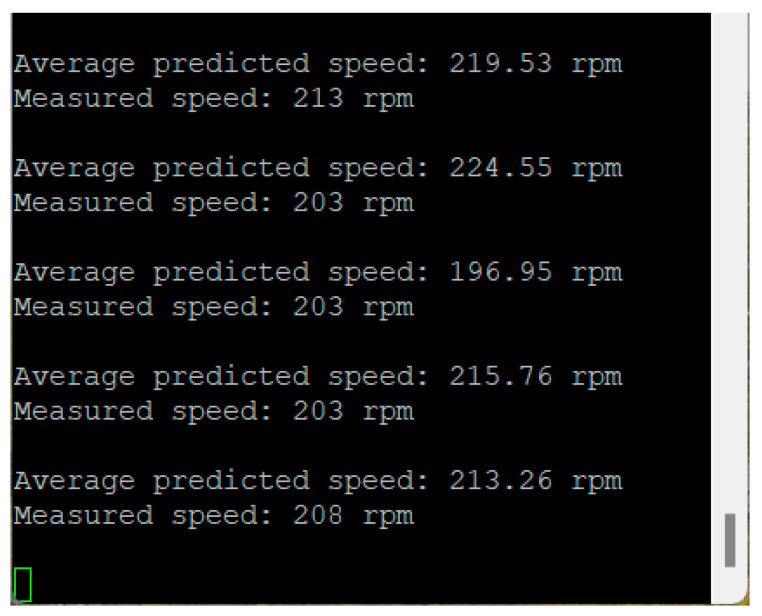
Serial port—speed prediction vs. measurements—200 rpm.

**Figure 9:**
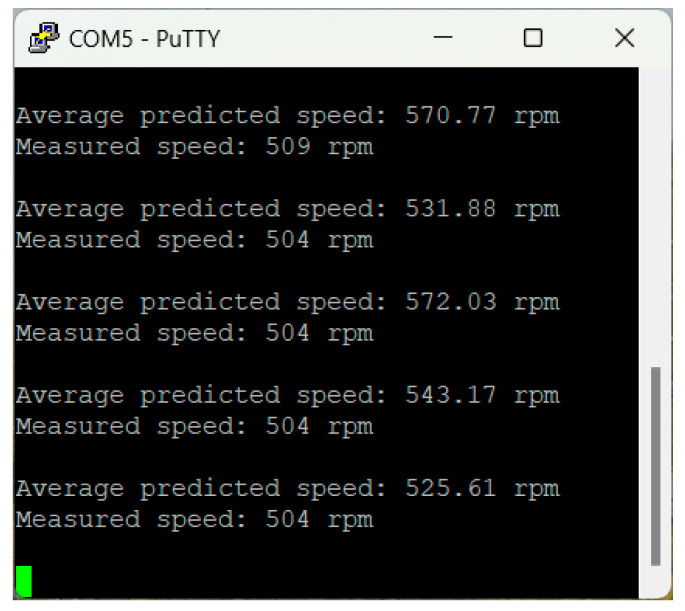
Serial port—speed prediction vs. measurements—500 rpm.

**Figure 10:**
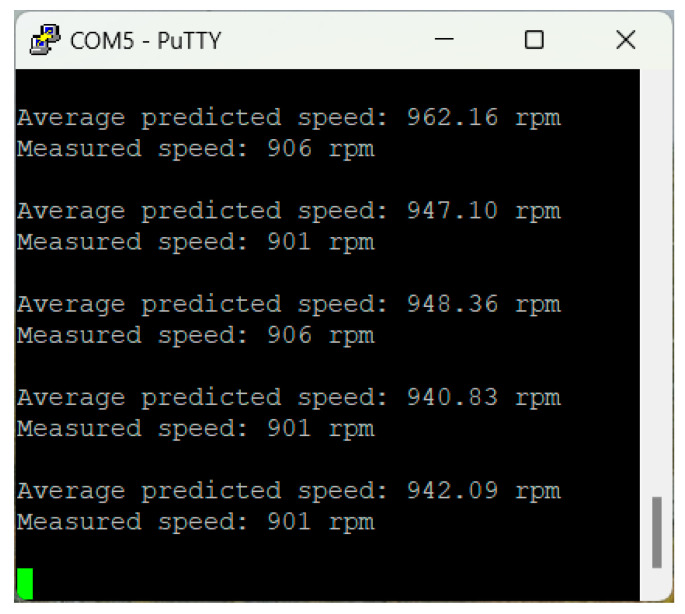
Serial port—speed prediction vs. measurements—900 rpm.

In the experimental activity, the CNN model achieved an overall Mean Absolute Error (MAE) of 42.1 rpm across the operational speed range of 200–1500 rpm. The detailed MAE for each speed category indicates consistent performance without any apparent trend across different speed ranges. Although the selected metric does not allow an evaluation of the sign of the speed predictions, it was observed that the model prevalently outputs higher speeds than the measured values (Figure 8, Figure 9 and Figure 10). This bias suggests the presence of underlying systematic factors that may require further investigation, with one possible solution being the expansion of the training dataset.

The experimental results were collected under various conditions, including different times of the day, to account for changing levels of ambient noise in order to evaluate the system’s robustness. The experimental activity was carried out in the labs of mechanics with common testing machines and mechanical equipment operated by personnel throughout the day and, therefore, has a level of background noise comparable to that of a standard industrial environment. The MAE result of 42.1 rpm indicates that the model maintained a reasonable accuracy, highlighting CNN’s ability to filter out irrelevant information, ensuring reliable performance in real-world industrial environments.

The Opta’s RAM usage is around 85%, indicating how effective optimization techniques, such as those presented in the previous section, are paramount for AI feasibility on edge devices.

However, an average MAE of 42.1 rpm significantly reduces the applicability of such a method in environments where precise speed measurements are required. However, the aim was not to prioritize accuracy but rather to investigate the workflow and tools at one’s disposal and the trade-offs that are necessary to deploy complex AI models on resource-constrained platforms in anticipation of further research in the field of predictive maintenance. This was a goal that was successfully met.

## 5. Conclusions

This study successfully demonstrated the feasibility of deploying a convolutional neural network model on the Finder Opta™ PLC for real-time rotational speed inference. The proof-of-concept achieved in this research allowed for the investigation of the available tools and required workflow, opening the door for future advancements in predictive maintenance on edge devices.

The Finder Opta™ PLC, thanks to its integration with the Arduino ecosystem, proved capable of handling the computational demands of the CNN model after appropriate optimization and quantization. This accomplishment highlights the potential of leveraging existing industrial hardware to incorporate advanced AI functionalities without substantial infrastructural changes.

The model’s consistent performance under varying noise conditions attests to its robustness and reliability, which are crucial for real-world industrial applications. However, its generalizability to other systems or varied industrial environments has not been fully assessed.

Deploying machine learning models on existing PLCs like the Finder Opta™ offers a cost-effective alternative to traditional centralized cloud-based solutions. The minimal additional infrastructure required and the ability to retrofit into existing production lines make this approach highly scalable. Smaller manufacturing plants, which may not have the budget or technical skills for extensive upgrades, can adopt this methodology to enhance their maintenance practices, as will be explored in more detail in subsequent studies, thereby improving access to advanced predictive maintenance and manufacturing technologies.

### Future Developments

To build upon the findings of this study, future developments will focus on expanding the applicability of the proposed solution to monitor the health of mechanical machinery in broader predictive maintenance frameworks. Specifically, this will involve leveraging acoustic data to detect anomalous sounds indicative of wear, misalignment, or other potential faults. To achieve this, research will investigate advanced signal processing techniques and machine learning methods to enhance the system’s ability to distinguish between operational noise and early-stage fault signals. Efforts will also aim at reducing prediction errors and mitigating the effects of environmental disturbances, such as varying background noise levels.

For instance, processing the raw audio signal with the Wavelet transform instead of the FFT could provide richer time–frequency domain features, particularly for non-stationary signals such as acoustic data from rotating machinery. The Wavelet transform’s ability to capture localized frequency components could improve the model’s ability to detect subtle variations, potentially enhancing the accuracy of predictions or enabling the system to detect early-stage anomalies. Additionally, examining alternative machine learning models, such as recurrent neural networks (RNNs) for sequential data or hybrid approaches combining feature extraction with traditional classifiers, may yield models better suited for varied predictive maintenance tasks. By addressing these aspects, future studies could extend the applicability of this work, offering insights into optimizing edge AI applications across a broader spectrum of industrial applications.

## Figures and Tables

**Table 1:** Experimental MAE per speed category.

Speed Label [rpm]	MAE [rpm]	Speed Label [rpm]	MAE [rpm]
200	56.1	900	34.5
250	29.9	950	62.1
300	35.8	1000	36.5
350	28.3	1050	44.0
400	53.2	1100	59.7
450	22.6	1150	42.2
500	36.1	1200	45.0
550	46.5	1250	59.1
600	60.5	1300	41.2
650	29.5	1350	42.0
700	33.4	1400	47.7
750	36.3	1450	40.9
800	45.1	1500	36.3
850	32.2		

**Table 2:** Overall Experimental MAE Result.

Overall Mean Absolute Error (MAE)	42.1 rpm

## Data Availability

The original contributions presented in this study are included in the article. Further inquiries can be directed to the corresponding author.
